# Efficacy of a novel hemostatic adhesive powder in patients with upper gastrointestinal tumor bleeding

**DOI:** 10.1186/s12876-021-01611-0

**Published:** 2021-01-28

**Authors:** Jongbeom Shin, Boram Cha, Jin-Seok Park, Weonjin Ko, Kye Sook Kwon, Jin-Woo Lee, Hyung Kil Kim, Yong Woon Shin

**Affiliations:** Department of Internal Medicine, Inha University Hospital, Inha University School of Medicine, 27 Inhang-ro, Jung-gu, Incheon, 22332 Republic of Korea

**Keywords:** Tumor bleeding, Salvage therapy, Hemostatic powder

## Abstract

**Background:**

Gastrointestinal tumor bleeding remains a clinical challenge because it is difficult to treat with conventional endoscopic hemostatic options. Recently, an endoscopic hemostatic powder (UI-EWD) was developed and reported to provide effective control of upper gastrointestinal bleeding. The aim of current study was to evaluate the feasibility and efficacy of this novel hemostatic powder in tumor bleeding.

**Methods:**

A total of 41 consecutive patients with upper gastrointestinal tumor bleeding were included. UI-EWD was applied in all patients as an auxiliary hemostatic method as a salvage therapy or monotherapy during endoscopic treatment. Hemostasis success rates, adverse event related to UI-EWD, and rates of re-bleeding were evaluated.

**Results:**

In all cases, UI-EWD application was successful at tumor bleeding sites. Immediate hemostasis occurred in 40/41 (97.5%) patients, and re-bleeding within 28 days occurred in 10 of 40 (22.5%) patients that achieved initial hemostasis. The success rate of immediate hemostasis for UI-EWD monotherapy was 100% (23/23). The re-bleeding rate at 28 days after UI-EWD monotherapy was 26.1% (6/23). No adverse events associated with UI-EWD application were encountered.

**Conclusions:**

The success rate of UI-EWD for immediate hemostasis in cases of GI tumor bleeding was excellent and UI-EWD produced promising results with respect to the prevention of re-bleeding. Based on these results, we suggest that UI-EWD be considered an effective salvage therapy or even monotherapy for GI tumor bleeding.

## Background

Tumor related gastrointestinal bleeding (GIB) is not a rare condition and is responsible for up to 5% of GI bleeding cases [1–3]. GIB related tumors occur in the settings of primary gastrointestinal (GI) tumor, metastatic disease to the GI tract, or locally invasive tumor [4], and are commonly treated endoscopically by injection or using thermal or mechanical devices [5]. However, the endoscopic management of tumor bleeding is often clinically challenging due to poor patient clinical status and difficulties associated with the accessibility and extent of bleeding lesions. Notably, the immediate endoscopic hemostasis rate of tumor bleeding has been reported to be as low as 30% and the re-bleeding rate as high as 40% [6]. Therefore, further developments in the endoscopic methods of hemostasis are warranted.

Hemostatic powders have recently been studied for use in endoscopic applications and been reported to provide excellent immediate hemostatic rates (93–98%) for GIB and tumor bleeding (80–100%) [7–9]. However, reported re-bleeding rates (33–49%) [8, 10, 11] were high, and the technique was found to present technical challenges during application, such as clogging of the delivery catheter and obscuring of target lesions after failed application [12]. Recently, a newly developed adhesive hemostatic powder (UI-EWD, Nextbiomedical, Incheon, Republic of Korea) was reported to be effective in refractory UGIB and non-variceal bleeding [13, 14].

UI-EWD is composed of a biocompatible natural polymer produced using aldehyde dextran and succinic acid modified ε‐poly (l‐lysine), which immediately converts to a highly adhesive hydrogel in the presence of water. Hydrogels are easily formed by the reaction between aldehyde (aldehyde dextran) and amino (ε-poly) groups, which results in Schiff base formation, multiple crosslinks, and high adhesive strength. A subsequent coating process is used to modify the water absorbing capacity of UI-EWD using a fluidized bed granulator and liquid coating materials. This coating technology allows the UI-EWD to be delivered to sites of bleeding without catheter clogging.

The aim of the current study was to evaluate the hemostatic effect of UI-EWD for the treatment of tumor bleeding. In addition, we also evaluated re-bleeding rates after UI-EWD application.

## Methods

### Study design and study population

**Fig. 1:**
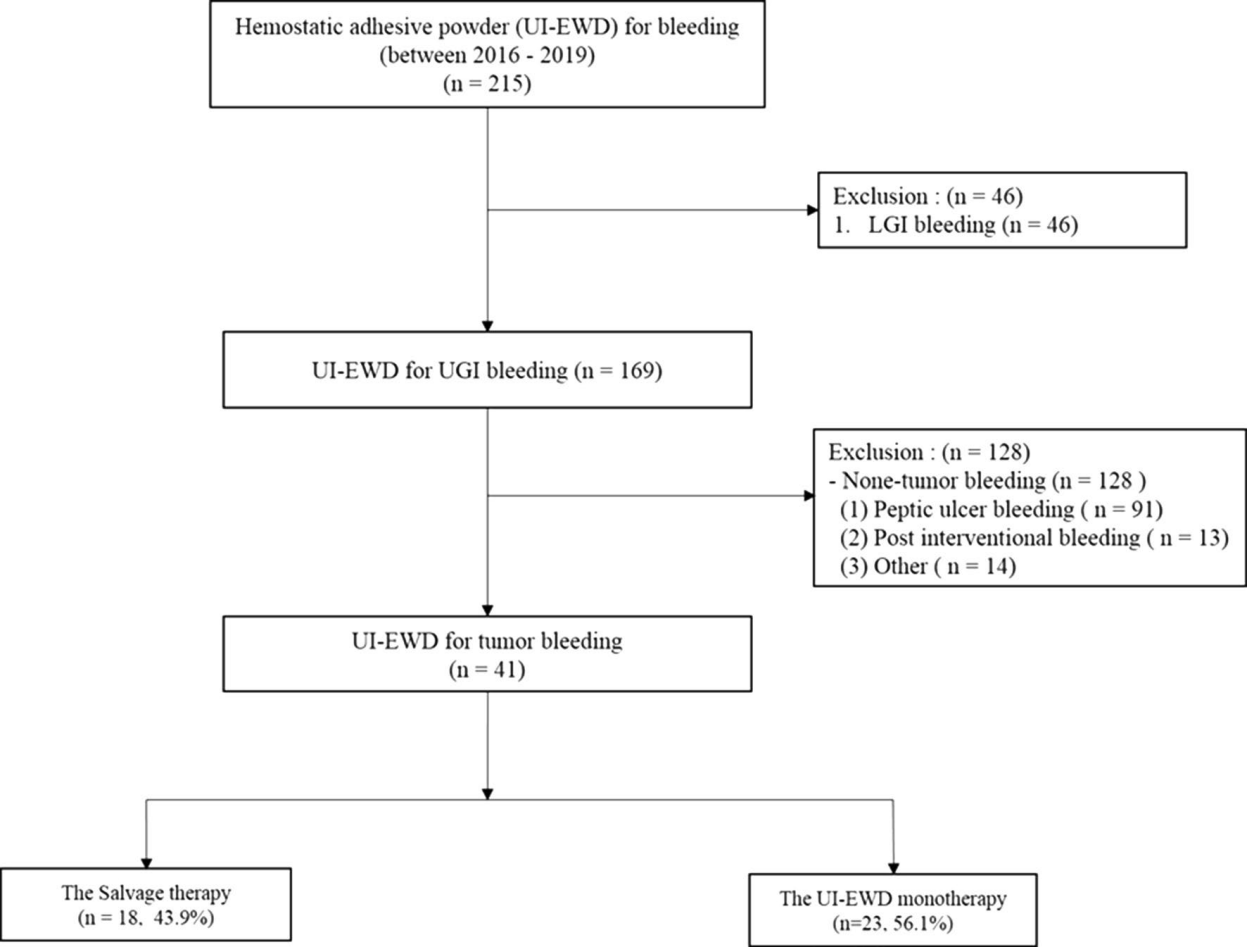
Patient selection flowsheet

Patients treated with UI-EWD for upper gastrointestinal (UGI) tumor bleeding during the period from January 2016 to December 2019 at Inha university hospital were enrolled. Patients were retrospectively selected from established prospective registries using the following inclusion criteria.: (1) age > 18 years at time of treatment; (2) signs of acute GI bleeding (e.g., fresh blood, blood clots in vomit, and/or melena); (3) tumor bleeding from gastric cancer, lymphoma, or metastatic cancer (4) receipt of endoscopic hemostasis using UI-EWD. Patients that met the following criteria were excluded: (1) low GI tract bleeding; (2) non-tumor GI bleeding caused by peptic ulcer, post-endoscopic therapy, varix, or others; (3) pregnancy or suspected pregnancy at time of treatment; and (4) receipt of another endoscopic or surgical treatment within 30 days prior to UI-EWD application. Forty-one patients met the study criteria (Fig. 1). Patient medical records were reviewed, and information on clinical characteristics, bleeding, clinical outcomes (including immediate hemostasis success and re-bleeding rate), and UI-EWD-related complications were collected. The study protocol was approved by the Institutional Review Board of our institution (INHAUH 2020-03-010).

### Endoscopic procedures

**Fig. 2:**
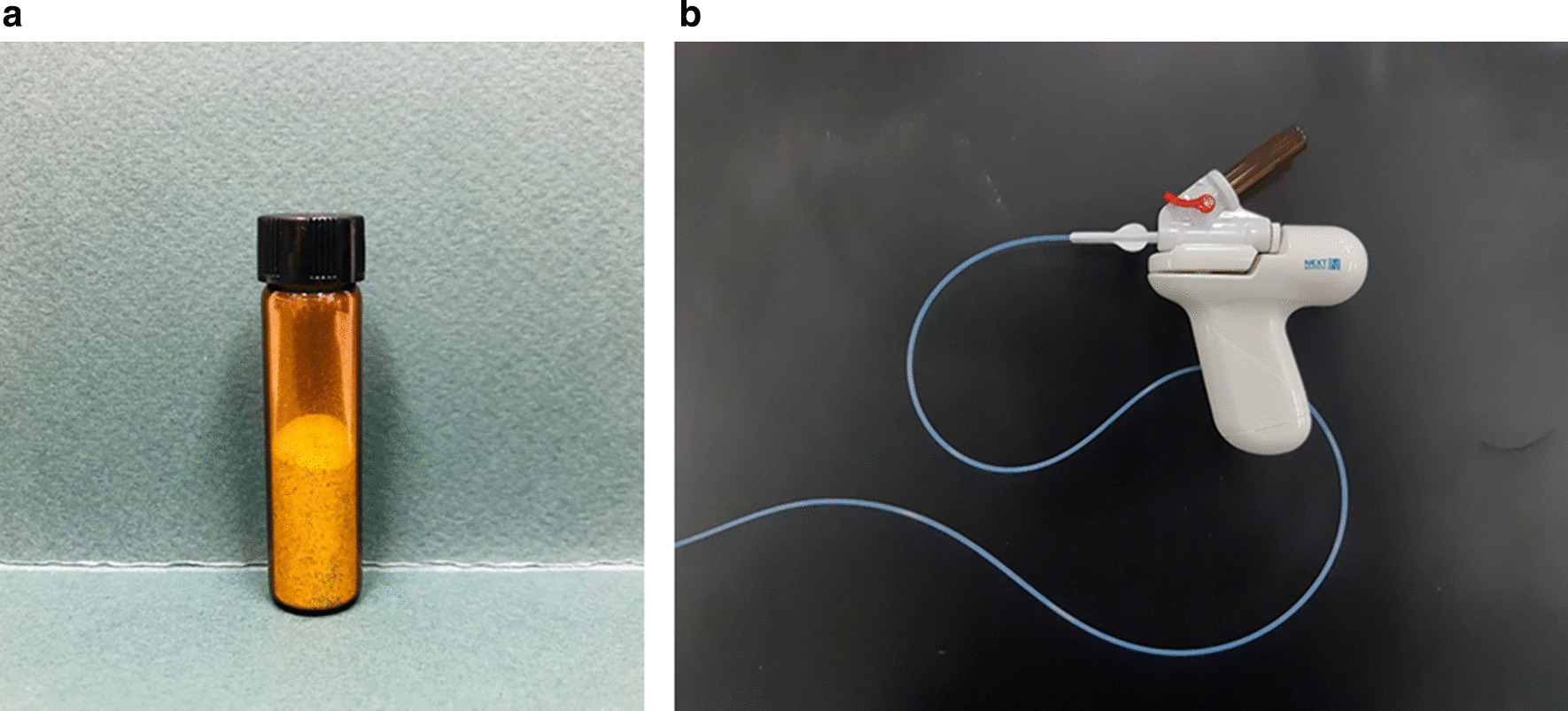
Images of UI-EWD (**a**) and the spraying device (**b**)

**Fig. 3:**
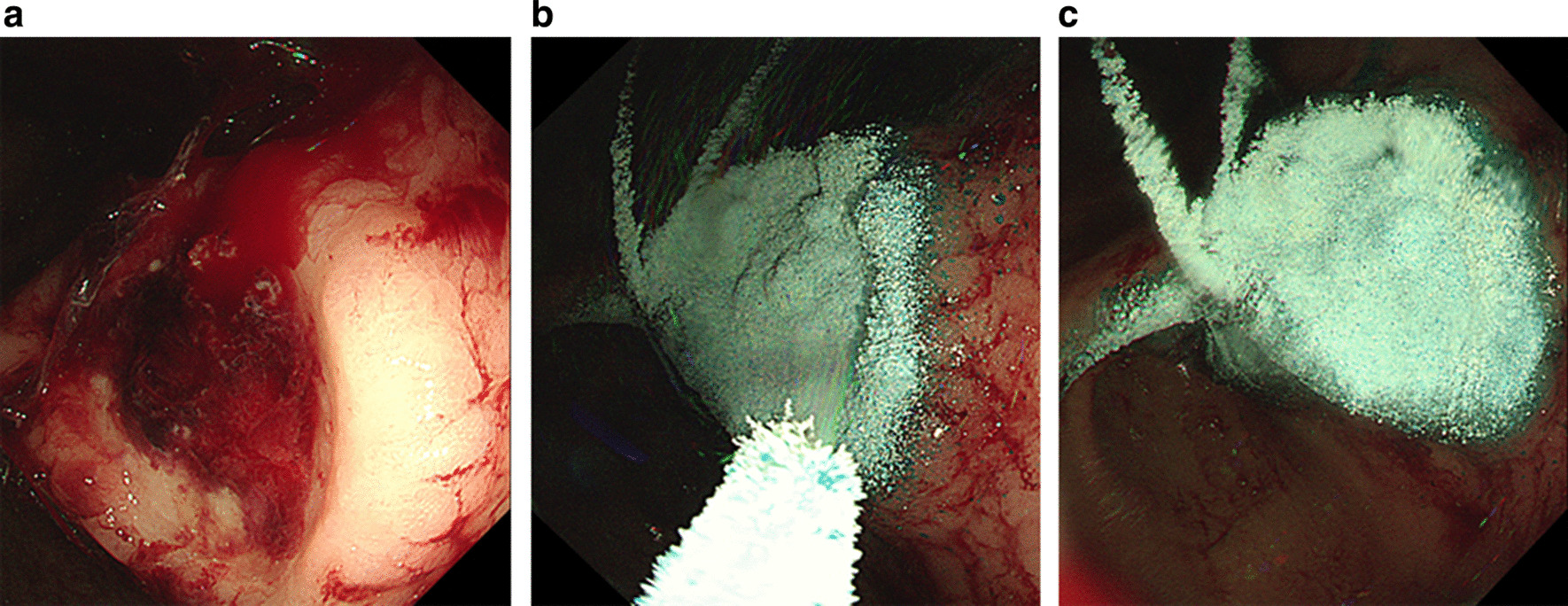
The endoscopic images of UI-EWD application for upper gastrointestinal tumor bleeding. **a** The patient had Forrest-Ib bleeding from gastric antrum due to advanced gastric cancer that could not be controlled by thermal therapy. **b** Application of UI-EWD at the bleeding site. **c** Five minutes after application, the UI-EWD was firmly attached at the tumor bleeding site without any sign of bleeding

UI-EWD was applied to tumor bleeding sites using a conventional endoscope (GIF-HQ290, Olympus, Tokyo, Japan) by experienced (6–25 years) endoscopists. In principle, UI-EWD was used as a salvage therapy when bleeding was sustained despite treatment with conventional hemostatic modalities, such as thermal therapy (heater probe, monopolar probe, and/or argon-plasma-coagulation) and/or clipping (hemoclips). In this study, conventional hemostatic modalities failed to achieve immediate hemostasis, and UI-EWD was applied immediately by the same endoscopist. However, when lesions were too large to address using a conventional modality, UI-EWD was applied as a monotherapy. UI-EWD was applied to tumor bleeding sites using the UI-EWD delivery system (Fig. 2) under direct endoscopic vision until lesions were completely covered (Fig. 3). UI-EWD was applied in bursts, and the maximum amount of powder released per lesion was 6 g.

### Definition & clinical outcomes

Successful immediate hemostasis was defined as hemostasis within 5 min of powder application as determined by visual inspection. Immediate hemostasis failure was defined as the need for an additional treatment modality after first applying UI-EWD due to persistent bleeding for more than 5 min. In cases of immediate hemostasis failure, the application of UI-EWD was repeated or another hemostatic modality, such as emergency operation or vascular embolization were applied. The additional treatments applied and types of additional treatments were determined at the endoscopist discretion. Re-bleeding was defined as clinical evidence of bleeding (e.g., melena or hematemesis) with an associated reduction of 2 g/dL in hemoglobin within 30 days of the endoscopic procedure. When re-bleeding was suspected, further endoscopic evaluation was performed to confirm its status. We reviewed all medical records to assess adverse events associated with UI-EWD, such as newly developed symptoms (e.g., abdominal pain, nausea, and vomiting), changes in vital signs, and laboratory test abnormalities.

### Statistical analysis

The clinical characteristics of the study subjects are expressed as medians (ranges) for continuous variables and numbers (percentages) for categorical variables. Overall re-bleeding rates and cumulative survival rates were estimated using the Kaplan–Meier method. The statistical analysis was performed using SPSS v25.0 (IBM Corp. Released 2017. IBM SPSS Statistics for Windows, Version 25.0. Armonk, NY: IBM Corp).

## Results

### Baseline patient characteristics

During the 3-year study period, 41 patients with upper GI tumor bleeding were treated using UI-EWD by experienced endoscopists. Median patient age was 74 years (range 39–88 years) and the majority were male (n = 31, 75.6%). The most frequent histologic type was adenocarcinoma (n = 33, 80.5%). Most patients had an ASA score of ≥ 3 (n = 32, 78%), and the most common comorbidity was HTN (n = 18, 43.9%). Eighteen patients (43.9%) received antithrombotic or anticoagulation therapy, and 73.2% (n = 30) had advanced disease (tumor stage IV). Median follow up duration was 107 days (range 7–956 days). Baseline patient characteristics are shown in Table 1.

**Table 1:** Baseline clinical characteristics of study subjects

Variables	Total enrolled patients (n = 41)	UI-EWD monotherapy (n = 23)
Age (year)^§^	74 (39–88)	76 (39–88)
Gender (Male), n (%)	31 (75.6)	32 (74.4)
*The tumor pathology, n (%)*		
Adenocarcinoma	33 (80.5)	17 (73.9)
Squamous carcinoma	2 (4.9)	0 (0.0)
GIST	5 (12.2)	4 (17.4)
Lymphoma	1 (2.4)	2 (8.7)
*Tumor stage, n (%)*		
1	3 (7.3)	2 (7.0)
2	2 (4.9)	0 (9.3)
3	6 (14.6)	3 (16.3)
4	30 (73.2)	18 (67.4)
*ASA score, (%)*		
1	2 (4.9)	1 (4.3)
2	7 (17.1)	4 (17.4)
3	18 (43.9)	10 (43.5)
4	13 (31.7)	7 (30.4)
5	1 (2.4)	1 (4.3)
*Comorbidity, n (%)*		
HTN	18 (43.9)	12 (47.8)
DM	12 (29.3)	5 (21.7)
Cardiovascular	13 (31.7)	7 (30.4)
CKD	4 (9.8)	2 (8.7)
Systolic blood pressure (mmHg)^§^	108 (74–162)	108 (74–139)
Diastolic blood pressure (mmHg)^§^	59 (30–96)	59 (30–91)
Heart rate (per min)^§^	92 (51–177)	84 (51–177)
Hb (g/dL)^§^	6.7 (3.7–14.0)	6.7 (3.7–14.0)
Follow up duration (day) ^§^	107 (7–956)	57 (7–607)

ASA, American society of anesthesiologist; CKD, chronic kidney disease; DM, diabetes mellitus; GIST, gastrointestinal stromal tumor; Hb, hemoglobin; HTN, hypertension

^§^, median (range)

### Bleeding characteristics

The stomach was the most frequent bleeding location (33/41; 81.4%), and of these cases, two were located in fundus or cardia, 16 in the stomach body, and 15 in the stomach antrum. In 5 of 41 patients (12.2%), bleeding was located in duodenum, and all were located in the 2nd portion of duodenum. Three bleedings (7.3%) were in the mid to distal esophagus. The majority of bleedings were classified as Forrest Ib (38 of 41 patients, 92.7%). The most frequently used conventional hemostasis modality was a thermal hemostatic grasper (9/18; 50.0%). The median tumor diameter was 5 cm (range 1–15 cm). In 23 of the 41 patients, UI-EWD was the only modality used to treat tumor bleeding. Table 2 summarizes the characteristics of tumor bleeding and includes etiologies and locations (Table 3).

**Table 2:** Bleeding characteristics of study subjects

Variables	Total enrolled patients (n = 41)	UI-EWD monotherapy (n = 23)
*Location of the tumor bleeding, n (%)*		
Esophagus	3 (7.3)	2 (8.7)
Stomach	33 (80.5)	20 (87.0)
Fundus and cardia	2 (4.9)	1 (4.3)
Body	16 (39.0)	9 (39.1)
Antrum	15 (36.6)	10 (43.6)
Duodenum	5 (12.2)	1 (4.3)
*Forrest classification, n (%)*		
Ia	3 (7.3)	0 (0.0)
Ib	38 (92.7)	23 (100.0)
Tumor size (cm)^§^	5 (1–15)	5 (1–17)
*The treatment modality, n (%)*		
UI-EWD only	23 (56.1)	23 (100.0)
Coagraspher with UI-EWD	9 (22.0)	
APC with UI-EWD	4 (9.8)	
Hemoclipping with UI-EWD	3 (7.3)	
Epinephrine injection with UI-EWD	2 (4.9)	

APC, argon plasma coagulation

^§^, median (range)

**Table 3:** The clinical outcomes of UI-EWD in tumor bleeding

Variables	Total enrolled patients (n = 41)	UI-EWD monotherapy (n = 23)
Success of Immediate hemostasis, n (%)	40 (97.5)	23 (100.0)
Cumulative re-bleeding, n (%)	9/40 (22.5)	6/23 (26.1)
At 7 day	3 (7.5)	1 (4.3)
At 28 days	9 (22.5)	6 (26.1)
At 180 days	9 (22.5)	6 (26.1)
Time to re-bleeding (day)^§^	10 (1–23)	12 (7–22)
RBC transfusion (unit)^§^	2 (0–6)	2 (0–6)
Hospital days^§^	10 (3–48)	10 (5–45)
*After failure of hemostasis, method for treatment, n (%)*		
Embolization	1 (100.0)	0 (0.0)
Surgery	0 (0.0)	0 (0.0)
Conservative management	0 (0.0)	0 (0.0)

RBC, red blood cell

^§^, median (range)

### Clinical outcomes

**Fig. 4:**
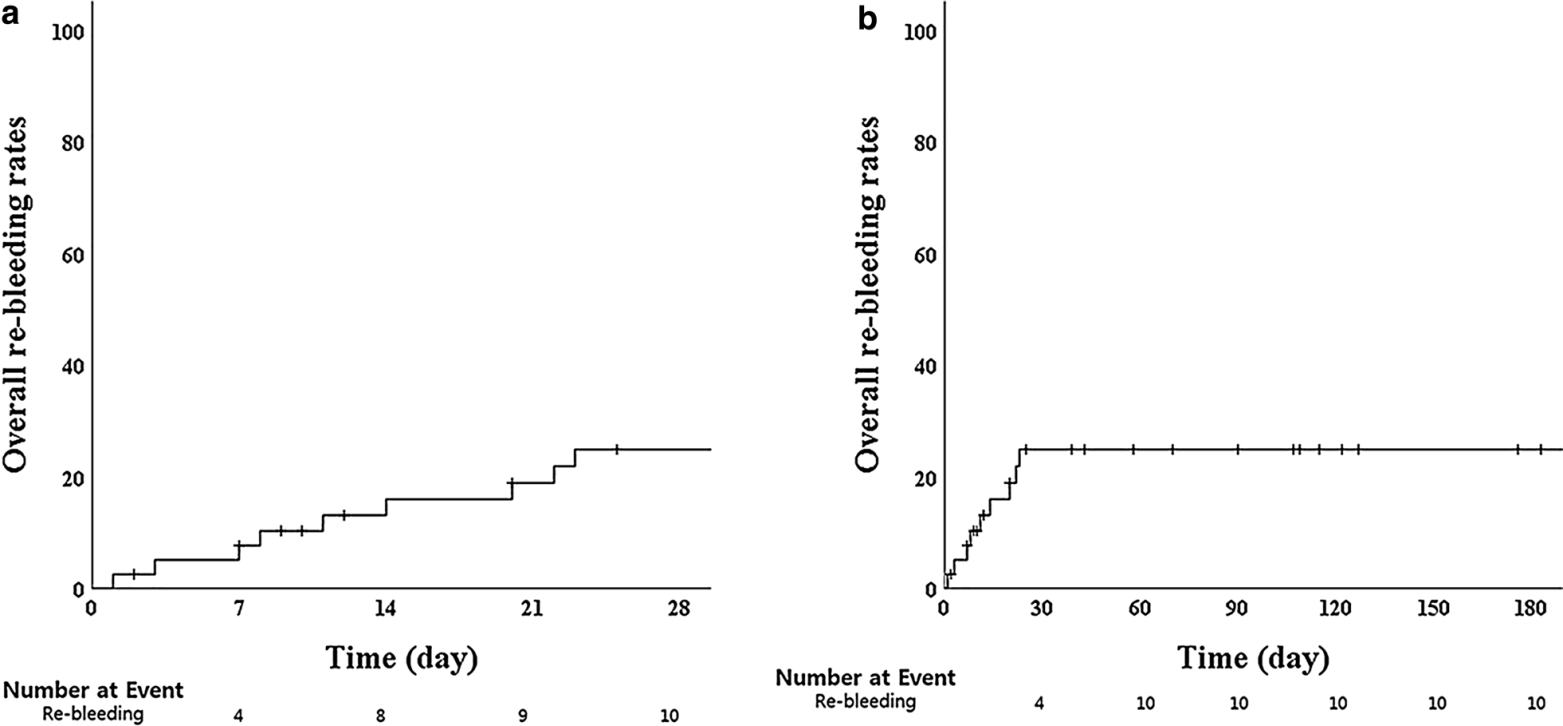
Overall re-bleeding rates at 28 (**a**) and 180 days (**b**) after the endoscopic procedures

Immediate hemostasis success was achieved in 40 of the 41 patients (97.5%), the single failure involved duodenal invasion of pancreatic cancer. In this patient, hemostasis was completed by arterial embolization. Overall re-bleeding rates at 7 and 28 days after hemostasis were 7.5% (3 of 40 patients) and 22.5% (9 of 40 patients), respectively. Median time to re-bleeding was 10 days (range 1–23 days). For UI-EWD monotherapy, the immediate hemostasis success rate was 100% (23/23), and overall re-bleeding rates at 7 and 29 days were 4.3% and 26.1%, respectively (Fig. 4). No adverse events such as infection, intestinal obstruction and perforation were associated with UI-EWD application.

## Discussion

We report the results of UI-EWD as a tumor bleeding therapy in 41 patients over a three-year period. In our cohort, UI-EWD was applied as a salvage therapy for tumor bleeding when conventional hemostasis was not sufficient or as a monotherapy in cases unsuitable for conventional hemostatic modalities. UI-EWD was found to have an excellent immediate hemostatic success rate (97.5%) and a low re-bleeding rate (7.5% and 22.5% on days 7 and 28, respectively). To the best of our knowledge, the present study is one of the largest to be conducted on the use of a hemostatic powder to treat GI tumor bleeding.

Tumor bleeding presents challenges in clinical settings because conventional endoscopic hemostasis is not always effective [15]. According to limited reports on the subject, the initial hemostasis success rate of conventional endoscopic therapy for tumor bleeding ranges from 31 ~ 40% and the short-term re-bleeding rate is about 80% [16, 17]. Hemostasis failure of conventional endoscopic modalities is associated with tumor size, friability, pathological angiogenesis, or tumor induced necrosis [18–20]. In addition, the acidic stomach environment and pancreatic juice can promote tumor bleeding because they dissolve blood clots and digest tumor tissues, which lack a protective barrier epithelium and mucous [16]. When endoscopic hemostasis fails, more invasive treatments, such as surgery or interventional angiography are required for hemostasis [20, 21]. However, surgery has a high mortality rate [20] and interventional angiography has a poor clinical success rate [22] and introduces the risk of complications such as ischemic organ damage [23]. Accordingly, there is need for a noble, effective endoscopic hemostatic modality for tumor bleeding. In the present study, UI-EWD was found to have an excellent immediate hemostasis success rate, which is in accord with previous findings [10, 24]. This high success rate can be explained by the hydrogel nature of UI-EWD. Usually, hemostasis is difficult to achieve at tumor bleeding sites by conventional hemostasis because bleeding surfaces are large with multiple bleeding points [25, 26]. The hydrogel formed by UI-EWD covers entire tumor bleeding surfaces and prevents contact with acidic media, pancreatic juice, and bile.

The relatively high re-bleeding rate (33–49%) [8, 10, 11] of tumors after immediate hemostasis presents a technical challenge to previously existing hemostatic powders. The 30-day re-bleeding rate in our study was 22.4%, which is lower than the re-bleeding rates of other commercially available hemostatic powders (47%) in high risk, non-variceal UGIB patients [8]. Furthermore, the 6-month cumulative survival rate obtained in our study was 73.2% (n = 30) and the follow up period was adequate (Table 1). We attribute this lower re-bleeding rate to strong tissue adhesion exhibited by UI-EWD, which remains well attached despite intestinal peristalsis. UI-EWD hydrogel has been reported to be present at 70.2% of sprayed bleeding sites by second-look endoscopy at 24 h [27]. Therefore, we believe UI-EWD provides much better attachment than other available hemostatic powders, which makes it a useful tool for the treatment of tumor bleeding and promising option in terms of reducing re-bleeding rates. In a retrospective case series on malignant GI bleeding, 30-day mortality rates of 14% to 48% were reported [18, 28, 29]. Re-bleeding is known to be associated with poor survival in patients with hemostasis [15, 30]. In the current study, two patients experienced re-bleeding and received additional UI-EWD treatment up to 3 times as bridge therapy. These patients eventually underwent chemotherapy and surgery and survived for more than a year. Therefore, we assume that UI-EWD can be considered a bridge therapy modality in selective patients even if re-bleeding occurs after immediate hemostasis of tumor bleeding has been achieved.

Because the purpose of endoscopic therapy in patients with tumor bleeding is salvage or bridge therapy, complications caused by the treatment modality during tumor bleeding control may adversely affect prognosis. In a previous study, perforation was reported to be a severe adverse event of other commercially available hemostatic powders [31], and there are also concerns about the risks of obstruction and systemic embolization [32]. Furthermore, perforations resulting from the use of other commercial hemostatic powders has been attributed to tissue rupture and perforation caused by high spraying pressures [31]. UI-EWD was specially designed to eliminate the effect high application pressures. No perforation or tissue damage associated with hemostatic powder application was observed in our cohort, and no other hemostatic powder related complications were observed.

Of the 41 patients included in this study, 18 patients (43.9%) were treated with UI-EWD as a salvage therapy (Fig. 1), and electrocoagulation was used in more than half of these patients. UI-EWD was applied as a monotherapy in 23 (56.1%) patients and immediate hemostasis success rates were similar in those treated with UI-EWD as a salvage therapy. Re-bleeding rates in these two groups at 7 and 28 days were 4.3% (n = 1) and 26.1% (n = 6), which was not inauspicious. On the other hand, Forrest Ib was the most frequent bleeding type in the monotherapy group. There was no case of Forrest type Ia. To more comprehensively assess the efficacy of UI-EWD monotherapy as a salvage therapy for tumor bleeding for different Forrest types a well-designed, large-scale prospective study is needed.

This current study has some limitations. First, the study is inherently limited by its retrospective design and lack of a control cohort. However, there are already many reported researches on conventional endoscopic hemostasis for tumor bleeding, this study shown the results that can be considered sufficiently effective. Second, the number of study subjects was relatively small. However, previous studies on the effects of hemostatic powders on tumor bleeding enrolled fewer than 20 patients [24]. In fact, the present study was conducted on a larger cohort and with longer follow up than previous studies on the topic. Third, tumor bleeding locations varied, but despite this heterogeneity, immediate hemostasis was successful in all by one patient, in who initial bleeding control failed. Furthermore, this result also suggests UI-EWD is applicable at any location in cases of upper GI tumor bleeding, and that its hemostatic effects are independent of location. Forth, we couldn’t figure out the possible reasons of re-bleeding after successful immediate hemostasis. There were only 9 patients in the re-bleeding group, and there was no significant difference in patients and bleeding characteristics between the group with or without re-bleeding. (Additional file 1: Table 1 and 2) A large-scale, well-designed randomized controlled study is needed to determine the possible factors of re-bleeding after immediate hemostasis.

## Conclusion

In conclusion, this single-arm study demonstrated UI-EWD was associated with excellent immediate hemostasis rate and safety profile as well as low rebleeding for upper GI tumor bleeding. Further studies are necessary to confirm its efficacy compared to conventional endoscopic modality.

## Supplementary Information

**Additional file 1: Table 1**. Baseline clinical characteristics of study subjects according to re-bleeding.** Table 2**. Bleeding characteristics of study subjects according to re-bleeding.

## Data Availability

The clinical datasets supporting the results of this article are available from the corresponding author upon reasonable request.

## Abbreviations

APC: Argon plasma coagulation
ASA: American society of anesthesiologist
CKD: Chronic kidney disease
DM: Diabetes mellitus
GI: Gastrointestinal
GIB: Gastrointestinal bleeding
HTN: Hypertension
Hb: Hemoglobin
LGI: Lower gastrointestinal
RBC: Red blood cell
UGI: Upper gastrointestinal
UGIB: Upper gastrointestinal bleeding

## References

[1] Allum WH, Brearley S, Wheatley KE (1990). Acute haemorrhage from gastric malignancy. Br J Surg.

[2] Loftus EV, Alexander GL, Ahlquist DA (1994). Endoscopic treatment of major bleeding from advanced gastroduodenal malignant lesions. Mayo Clin Proc.

[3] Sheibani S, Kim JJ, Chen B (2013). Natural history of acute upper GI bleeding due to tumours: short-term success and long-term recurrence with or without endoscopic therapy. Aliment Pharmacol Ther.

[4] Imbesi JJ (2005). Kurtz RC A multidisciplinary approach to gastrointestinal bleeding in cancer patients. J Support Oncol.

[5] Ofosu A, Ramai D, Latson W (2019). Endoscopic management of bleeding gastrointestinal tumors. Ann Gastroenterol.

[6] Kawabata H, Hitomi M (2019). Motoi S Management of Bleeding from Unresectable Gastric Cancer. Biomedicines.

[7] Yau AH, Ou G, Galorport C (2014). Safety and efficacy of Hemospray® in upper gastrointestinal bleeding. Can J Gastroenterol Hepatol.

[8] Chen YI, Barkun A (2015). Nolan S Hemostatic powder TC-325 in the management of upper and lower gastrointestinal bleeding: a two-year experience at a single institution. Endoscopy.

[9] Sulz MC, Frei R, Meyenberger C (2014). Routine use of Hemospray for gastrointestinal bleeding: prospective two-center experience in Switzerland. Endoscopy.

[10] Cahyadi O, Bauder M, Meier B (2017). Effectiveness of TC-325 (Hemospray) for treatment of diffuse or refractory upper gastrointestinal bleeding - a single center experience. Endosc Int Open.

[11] Haddara S, Jacques J, Lecleire S (2016). A novel hemostatic powder for upper gastrointestinal bleeding: a multicenter study (the "GRAPHE" registry). Endoscopy.

[12] Barkun A (2013). New topical hemostatic powders in endoscopy. Gastroenterol Hepatol (N Y).

[13] Park JS, Kim HK, Shin YW (2019). Novel hemostatic adhesive powder for nonvariceal upper gastrointestinal bleeding. Endosc Int Open.

[14] Park JS, Bang BW, Hong SJ (2019). Efficacy of a novel hemostatic adhesive powder in patients with refractory upper gastrointestinal bleeding: a pilot study. Endoscopy.

[15] Kim YI, Choi IJ, Cho SJ (2013). Outcome of endoscopic therapy for cancer bleeding in patients with unresectable gastric cancer. J Gastroenterol Hepatol.

[16] Adler DG, Leighton JA, Davila RE (2004). ASGE guideline: the role of endoscopy in acute non-variceal upper-GI hemorrhage. Gastrointest Endosc.

[17] Roberts SE, Button LA (2012). Williams JG Prognosis following upper gastrointestinal bleeding. PLoS ONE.

[18] Arena M, Masci E, Eusebi LH (2017). Hemospray for treatment of acute bleeding due to upper gastrointestinal tumours. Dig Liver Dis.

[19] Chen YI, Barkun AN, Soulellis C (2012). Use of the endoscopically applied hemostatic powder TC-325 in cancer-related upper GI hemorrhage: preliminary experience (with video). Gastrointest Endosc.

[20] Blackshaw GR, Stephens MR, Lewis WG (2004). Prognostic significance of acute presentation with emergency complications of gastric cancer. Gastric Cancer.

[21] Heller SJ, Tokar JL, Nguyen MT (2010). Management of bleeding GI tumors. Gastrointest Endosc.

[22] Park S, Shin JH, Gwon DI (2017). Transcatheter arterial embolization for gastrointestinal bleeding associated with gastric carcinoma: prognostic factors predicting successful hemostasis and survival. J Vasc Interv Radiol.

[23] Yap FY, Omene BO, Patel MN (2013). Transcatheter embolotherapy for gastrointestinal bleeding: a single center review of safety, efficacy, and clinical outcomes. Dig Dis Sci.

[24] Chen YI, Wyse J, Lu Y (2020). TC-325 hemostatic powder versus current standard of care in managing malignant GI bleeding: a pilot randomized clinical trial. Gastrointest Endosc.

[25] Savides TJ, Jensen DM, Cohen J (1996). Severe upper gastrointestinal tumor bleeding: endoscopic findings, treatment, and outcome. Endoscopy.

[26] Barkun AN, Moosavi S (2013). Martel M Topical hemostatic agents: a systematic review with particular emphasis on endoscopic application in GI bleeding. Gastrointest Endosc.

[27] Shin YW, Bang BW, Kwon K (2018). Endoscopic allpication of new hemostatic powder (UI-EWD®) in gastrointestinal bleeding. Endoscopy.

[28] Pittayanon R, Rerknimitr R (2018). Barkun A Prognostic factors affecting outcomes in patients with malignant GI bleeding treated with a novel endoscopically delivered hemostatic powder. Gastrointest Endosc.

[29] Meng ZW, Marr KJ, Mohamed R (2019). Long-term effectiveness, safety and mortality associated with the use of TC-325 for malignancy-related upper gastrointestinal bleeds: a multicentre retrospective study. J Can Assoc Gastroenterol.

[30] Kim YI, Choi IJ (2015). Endoscopic management of tumor bleeding from inoperable gastric cancer. Clin Endosc.

[31] Hagel AF, Albrecht H, Nägel A (2017). The application of hemospray in gastrointestinal bleeding during emergency endoscopy. Gastroenterol Res Pract.

[32] Chen YI, Barkun AN (2015). Hemostatic powders in gastrointestinal bleeding: a systematic review. Gastrointest Endosc Clin N Am.

