# Supplementary material for: Efficacy of a novel hemostatic adhesive powder in patients with upper gastrointestinal tumor bleeding
Source: BMC Gastroenterol. 2021 Jan 28;21:40. doi: 10.1186/s12876-021-01611-0 (PMC7842074; doi:10.1186/s12876-021-01611-0)
Supplement: Supplementary file 1 — Additional file 1: Table 1. Baseline clinical characteristics of study subjects according to re-bleeding. Table 2. Bleeding characteristics of study subjects according to re-bleeding. [file 12876_2021_1611_MOESM1_ESM.docx]

Supplementary

**Supplementary table 1. Baseline Clinical & Stent characteristics of study subjects**

| **Variables** | **Without**  **re-bleeding**  **(n=31)** | **Re-bleeding**  **(n=9)** | **P value^*^** |
| --- | --- | --- | --- |
| Age (year)^§^ | 73 (39-88) | 79 (56-85) | 0.311 |
| Gender (Male)^§^ | 23 (74.2) | 7 (77.8) | 1.000 |
| The tumor pathology, n (%) |  |  | 0.800 |
| Adenocarcinoma | 25 (80.6) | 8 (88.9) |  |
| Squamous carcinoma | 2 (6.5) | 0 |  |
| GIST | 4 (12.9) | 1 (11.1) |  |
| Tumor stage, n (%) |  |  | 0.586 |
| 1 | 3 (9.7) | 0 |  |
| 2 | 2 (6.5) | 0 |  |
| 3 | 4 (12.9) | 2 (6.5) |  |
| 4 | 23 (74.2) | 7 (22.6) |  |
| ASA score, (%) |  |  | 0.385 |
| 1 | 2 (6.5) | 0 |  |
| 2 | 7 (22.6) | 0 |  |
| 3 | 12 (38.7) | 6 (66.7) |  |
| 4 | 10 (32.3) | 3 (33.3) |  |
| 5 | 1 (3.2) | 0 |  |
| Comorbidity, n (%) |  |  | 0.570 |
| HTN | 13 (41.9) | 5 (55.6) | 0.456 |
| DM | 7 (22.6) | 5 (55.6) | 0.097 |
| Cardiovascular | 9 (29.0) | 4 (44.4) | 0.437 |
| CKD | 3 (9.7) | 1 (11.1) | 1.000 |
| Systolic blood pressure (mmHg)^§^ | 107 (74-151) | 108 (92-162) | 0.314 |
| Diastolic blood pressure (mmHg)^§^ | 56 (30-91) | 63 (56-96) | 0.562 |
| Heart rate (per min)^§^ | 87 (51-177) | 101 (65-123) | 0.595 |
| Hb (g/dL)^§^ | 6.9 (4.4-14.0) | 6.5 (3.7-8.8) | 0.645 |
| Follow up duration (day) ^§^ | 122 (7-956) | 50 (7-490) | 0.550 |

**Abbreviation**:; CKD, chronic kidney disease; DM, diabetes mellitus; GIST, gastrointestinal stromal tumor; Hb, hemoglobin; HTN, hypertension)

^§^, median (range)

^*,^ *P* values were calculated using the *t*-test or Fisher’s exact test between without re-bleeding and re-bleeding

**Supplementary table 2. Bleeding characteristics of study subjects**

| **Variables** | **Without**  **re-bleeding**  **(n=31)** | **Re-bleeding**  **(n=9)** | **P value**^*^ |
| --- | --- | --- | --- |
| Location of the tumor bleeding, n (%) |  |  | 0.768 |
| Esophagus | 3 (9.7) | 0 |  |
| Stomach | 24 (77.4) | 8 (88.9) |  |
| Fundus and cardia | 1 (3.2) | 1 (11.1) |  |
| Body | 12 (38.7) | 4 (44.4) |  |
| Antrum | 11 (35.5) | 3 (33.3) |  |
| Duodenum | 4 (12.9) | 1 (11.1) |  |
| Forrest classification, n (%) |  |  | 1.000 |
| Ia | 3 (9.7) | 0 |  |
| Ib | 28 (90.3) | 9 (100.0) |  |
| Tumor size (cm)^§^ | 5 (1-15) | 4 (2-12) | 0.580 |
| The treatment modality, n (%) |  |  | 0.227 |
| UI-EWD only | 17 (54.8) | 6 (66.7) |  |
| Coagraspher with UI-EWD | 8 (25.8) | 1 (11.1) |  |
| APC with UI-EWD | 2 (6.5) | 2 (22.2) |  |
| Hemoclipping with UI-EWD | 3 (9.7) | 0 (0.0) |  |
| Epinephrine injection with UI-EWD | 2 (6.5) | 0 (0.0) |  |

**Abbreviation**: APC, argon plasma coagulation

^§^, median (range)

^*,^ *P* values were calculated using the *t*-test or Fisher’s exact test between without re-bleeding and re-bleeding
